# In silico designed novel multi-epitope mRNA vaccines against *Brucella* by targeting extracellular protein BtuB and LptD

**DOI:** 10.1038/s41598-024-57793-6

**Published:** 2024-03-27

**Authors:** Juan Shi, Yuejie Zhu, Zhengwei Yin, Yueyue He, Yujiao Li, Gulishati Haimiti, Xinru Xie, Ce Niu, Wenhong Guo, Fengbo Zhang

**Affiliations:** 1 Department of Clinical Laboratory, The First Affiliated Hospital of Xinjiang Medical University, No. 393, Xinyi Road, Urumqi, 830011 Xinjiang China; 2 Department of Reproductive Assistance, Center forReproductive Medicine, The First Affiliated Hospital of Xinjiang Medical University, No. 393, Xinyi Road, Urumqi, 830011 Xinjiang China; 3 Department of Immunology, School of Basic Medical Sciences, Xinjiang Medical University, No. 393, Xinyi Road, Urumqi, 830011 Xinjiang China; 4 Department of Blood Transfusion, First Affiliated Hospital of Xinjiang Medical University, No. 393, Xinyi Road, Urumqi, 830011 Xinjiang China; 5 State Key Laboratory of Pathogenesis, Prevention, Treatment of Central Asian High Incidence Diseases, the First Affiliated Hospital of Xinjiang Medical University, No. 393, Xinyi Road, Urumqi, 830011 China

**Keywords:** Immunology, Microbiology

## Abstract

*Brucella*, a gram-negative intracellular bacterium, causing Brucellosis, a zoonotic disease with a range of clinical manifestations, from asymptomatic to fever, fatigue, loss of appetite, joint and muscle pain, and back pain, severe patients have developed serious diseases affecting various organs. The mRNA vaccine is an innovative type of vaccine that is anticipated to supplant traditional vaccines. It is widely utilized for preventing viral infections and for tumor immunotherapy. However, research regarding its effectiveness in preventing bacterial infections is limited. In this study, we analyzed the epitopes of two proteins of *brucella*, the TonB-dependent outer membrane receptor BtuB and the LPS assembly protein LptD, which is involved in nutrient transport and LPS synthesis in *Brucella*. In order to effectively stimulate cellular and humoral immunity, we utilize a range of immunoinformatics tools such as VaxiJen, AllergenFPv.1.0 and SignalP 5.0 to design proteins. Finally, five cytotoxic T lymphocyte (CTL) cell epitopes, ten helper T lymphocyte (HTL) cell epitopes, and eight B cell epitopes were selected to construct the vaccine. Computer simulations are also used to verify the immune response of the vaccine. The codon optimization, in silico cloning showed that the vaccine can efficiently transcript and translate in E. coli. The secondary structure of mRNA vaccines and the secondary and tertiary structures of vaccine peptides were predicted and then docked with TLR-4. Finally, the stability of the developed vaccine was confirmed through molecular dynamics simulation. These analyses showed that the design the multi-epitope mRNA vaccine could potentially target extracellular protein of prevalent *Brucella*, which provided novel strategies for developing the vaccine.

## Introduction

Brucellosis is a zoonotic bacterial infection caused by *Brucella*^1^.This gram-negative coccus lacks flagella, does not produce spores, and grows slowly^2^. The World Health Organization(WHO) reports more than 500,000 new cases of human brucellosis annually^3^.At the same time, the WHO considers brucellosis to be one of the seven neglected zoonotic diseases^4^. It poses a threat to public health in the Mediterranean region, the Middle East, Central Asia, Southeast Asia, sub-Saharan Africa, and some parts of Latin America^5^. In 2019, there were 44,036 confirmed cases, resulting in an incidence rate of 3.2513 per 100,000 people in China ^6^.There are six common species of *Brucella*: *Brucella melitensis, Brucella abortus, Brucella ovis, Brucella suis, Brucella canis, and Brucella neotomae*^7^. The most virulent causes of human infection are *Brucella melitensis, Brucella abortus, Brucella suis, and Brucella canis*^8^.

*Brucella* can enter the body through multiple pathways owing to its potent pathogenicity and subsequently generate endotoxin, precipitating bacteremia and toxemia^9^. *Brucella* is able to survive in the human immune system is attributed to its array of invasive virulence factors and diverse strategies for evading cellular immunity, allowing it to replicate within intracellular niches^10^. Patients with brucellosis present a spectrum of symptoms, which can vary from being asymptomatic to experiencing fever, fatigue, loss of appetite, and joint, muscle, and back pain^11^.Severe individuals may manifest multiorgan dysfunction^10^. However, the increasing drug resistance rate of *Brucella* brought great challenges for clinical treatment^12,13^. Therefore, it is necessary to develop a new therapeutic approach against *Brucella* in early prevention. Vaccines are one of the most important tools in public health and play an important role in preventing and controlling infectious diseases^14^. Unfortunately, a vaccine for brucellosis in human beings has not been approved yet^15^ . Several types of vaccines are presently under development to address brucellosis, such as attenuated live, multi-epitope, DNA, recombinant protein, and mRNA vaccines. The mRNA vaccines have been applied in Achromobacter xylosoxidans^16^ 、Citrobacter freundii (C. freundii)^17^ and so on. mRNA vaccines are an emerging approach to vaccine development, encoding only the target antigen to enhance the safety and efficacy of the vaccine^18^.

In order to expand the scope of vaccine application and provide cross-protection against *Brucella melitensis, Brucella abortus,* and *Brucella suis,* we selected the BtuB and LptD proteins, which are present in all three strains^19^. The outer membrane proteins BtuB, which transport vitamin B12, has been proved to have good immunogenicity in previous experiments and is suitable as a vaccine design candidate protein^20,21^. LptD is one of the outer membrane proteins of *Brucella* and has good immunogenicity^19^. The difference is that LptD is to transport lipopolysaccharides (LPS) which lead to the occurrence of an inflammatory response^22^. Therefore, vaccines based on BtuB and LptD may reduce the inflammatory reaction and slow down the progression of the disease.

In this study, we will design the mRNA vaccine based on LptD and BtuB. The immunological characteristics of the selected protein were predicted using an online tool. The prediction of cytotoxic T lymphocyte (CTL) epitopes, helper T lymphocyte (HTL) epitopes, and B cell epitopes was conducted. After predicting T-cell and B-cell epitopes, it is essential to identify antigenic, non-allergenic and non-toxic epitopes for subsequent construction of the vaccine^23^.Additionally, we will conduct molecular docking with MHC alleles to further explore their interactions. Finally, the mRNA vaccine was developed and its physical and chemical properties were assessed. The Immune simulation was then conducted. The nucleotide sequence of the vaccine was optimized through codon analysis, followed by in silico cloning and simulated gel electrophoresis. The secondary structure of the constructed mRNA vaccine was analyzed, along with the secondary and tertiary structures of the multi-epitope fusion proteins. The mRNA vaccine was also docked with TLR-4 molecules. To assess the stability of atoms and molecules in the vaccine, a molecular dynamics model was used.

## Results

### Physicochemical properties of proteins

The protein sequences of LptD and BtuB obtained from NCBI will be included in the supplementary information. Conducting online software analysis, it has been confirmed that BtuB and LptD have antigenicity scores of 0.811642 and 0.860866, respectively. With both surpassing the 0.5 threshold and demonstrating strong antigenicity. Additionally, the AlgPred server has indicated that the BtuB and LptD proteins are likely non-allergenic. The amino acid number of BtuB and LptD are 620aa and 792aa respectively. The molecular weight of BtuB and LptD are 66,892.00 and 88553.50 respectively. Both BtuB and LptD are outer membrane proteins. Further predictions regarding the bioinformatics analysis of these two proteins will be presented in Table 1.

**Table 1:** The list of the Basic structure and physicochemical properties of amino acids.

	BTuB	LptD
Number of amino acids	620	792
Antigenicity	0.811642	0.860866
Instability index	37.48	31.96
Grand average of hydropathicity(GRAVY)	− 0.334	− 0.421
Theoretical PI	5.26	5.37
Molecular weight	66,892.00	88,553.50
Allergenicity	Non-allergen	non-allergen
Subcellular localization	Cell outer membrane	Cell outer membrane

### Prediction and excision of signal peptides

**Figure 1:**
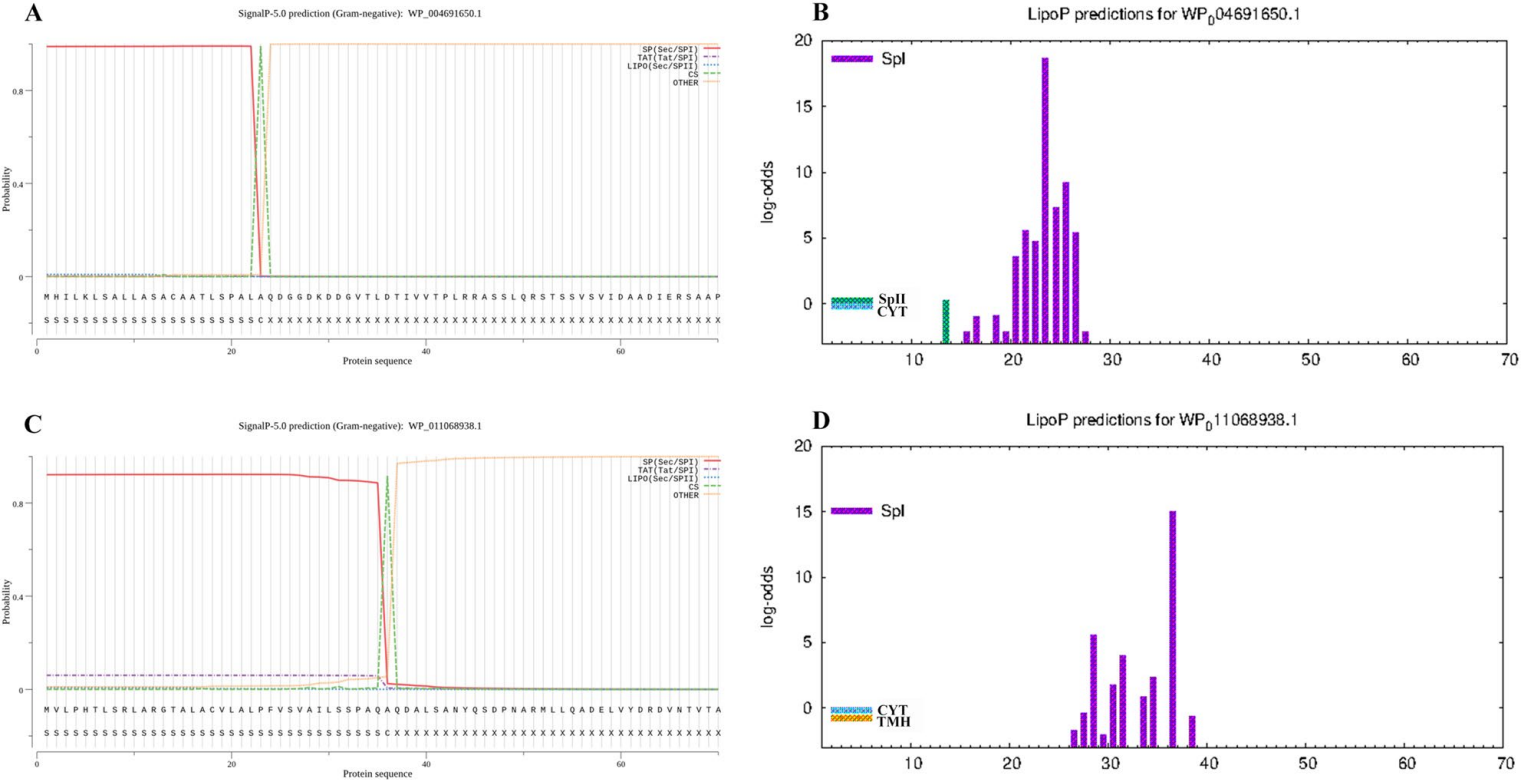
(**A**–**D**) Signal peptide of proteins using SignalP-5.0 and LiPOP1.0 analysis. SP (Sec/SPI) and Spl: type of signal peptide predicted; CS: the cleavage site; Other: the probability that the sequence does not have any kind of signal peptide. (**A**) The signal peptide prediction of BtuB by SignalP5.0. (**B**) The signal peptide prediction of BtuB by LiPOP1.0. (**C**) The signal peptide prediction of LptD by SignalP5.0.(**D**) The signal peptide prediction of LptD by LiPOP1.0.

SignalP 5.0 and LiPOP 1.0 were used to predict the signal peptides. The SignalP 5.0 server indicated that it is likely that the signal peptide of BtuB is located within the 1–24 peptide range (Fig. 1A). The LiPOP1.0 suggested that the 13–28 peptide may function as the signal peptide (Fig. 1B). According to the SignalP5.0 website, the 1–42 peptide of the LptD protein is highly likely to be the signal peptide (Fig. 1C). Additionally, the LiPOP 1.0 tool suggested that the signal peptide could be located between amino acids 26–39(Fig. 1D). Finally, we combined the results from two software programs and selected the peptide sequence that completely does not containa signal peptide in order to enhance the the effectiveness of the cell epitope. We choose the epitope after the 28 peptide of BtuB, and we choose the epitope after the 42 peptide of LptD.

### Prediction and assessment of B cell epitopes

After obtaining the predicted top ten epitopes from Ellipro and ABCpred respectively, we selected two cross-result epitopes. The next steps involved verifying their antigenicity, toxicity( See supplementary document 1), and allergenicity using VaxiJen, AllerTop V.2.0, and ToxinPred in turn. We screened all the predicted epitopes to determine whether they have homology with Homo sapiens (Taxid:9606), and we excluded peptides with E value less than 0.05. We selected two epitopes in BtuB protein, and six epitopes in LptD protein in Table 2.

**Table 2:** The list of the finally selected B cell epitopes and its antigenicity, toxicity and allergenicity.

	Start	End	Peptide	Score	Rank	Antigenicity	Toxicity	Allergenicity
BtuB	8	23	PECIESTNDEPENDEN	0.93	2	0.9364	Non-Toxin	NON- ALLERGEN
	462	477	QATASTSLDMALYQTR	0.92	3	0.9369	Non-Toxin	NON-ALLERGEN
LptD	756	771	MAYIQTRNPGDEKASH	0.93	3	0.9576	Non-Toxin	NON-ALLERGEN
	402	417	YSYTMPEPVYGGELNF	0.90	6	1.2036	Non-Toxin	NON-ALLERGEN
	342	357	TYEIQGYNAQTQVSKI	0.89	7	1.1514	Non-Toxin	NON-ALLERGEN
	486	501	MALRGDAIRVDTNFDP	0.88	8	1.3325	Non-Toxin	NON-ALLERGEN
	455	470	PGFSGTNLRFTSEAEW	0.88	8	0.9259	Non-Toxin	NON-ALLERGEN
	291	309	YLPSDKYEDEHPNDDSSR	0.817		0.7637	Non-Toxin	NON-ALLERGEN

### Prediction and assessment of T cell epitopes

We used the IEDB and the NetCTLpan1 server to predict CTL epitopes. We selected the top ten epitopes, two of which are consistent in both predictions. We also utilized IEDB and the NetMHC-IIpan-4.0 server to predict HTL epitopes. We selected the top ten epitopes, including two cross-result epitopes. It is worth mentioning that all the selected epitopes have been verified as antigenic, non-toxic, non-homologous, and non-allergenic. When predicting and evaluating CTL cell epitopes, five epitopes were selected: three from the BtuB protein and two from the LptD protein in Table 3. HTL cell epitopes were predicted and assessed. Six epitopes of the BtuB protein and four epitopes of the LptD protein were selected, as shown in Table 4. In the end, we selected 8 B cell epitopes, 5 CTL cell epitopes, and 10 HTC cell epitopes for the construction of subsequent mRNA vaccines.

**Table 3:** The list of the finally selected CTL cell epitopes and its antigenicity, toxicity and allergenicity.

	Allele	start	end	Peptide	Antigenicity	Toxicity	Allergenicity
BtuB	HLA-A*02:01	202	211	FLQGSFNFAL	0.8873	Non-Toxin	NON-ALLEGEN
	HLA-A*03:01	48	57	LQTYSGISVK	0.9789	Non-Toxin	NON-ALLEGEN
		67	76	NIYMRGMSSK	1.5162	Non-Toxin	NON-ALLEGEN
LptD	HLA-A*02:01	210	219	ALAPNYDLTL	1.3520	Non-Toxin	NON-ALLEGEN
		20	29	LVYDRDVNTV	0.9161	Non-Toxin	NON-ALLEGEN

**Table 4:** The list of the finally selected HTL cell epitopes and its antigenicity, toxicity and allergenicity.

	Allele	start	end	Peptide	Antigenicity	Toxicity	Allergenicity
BtuB	HLA-DRB1*07:01	421	435	GLNWQATASTSLDMA	1.2888	Non-Toxin	NON-ALLEGEN
		420	434	VGLNWQATASTSLDM	1.4454	Non-Toxin	NON-ALLEGEN
		419	433	EVGLNWQATASTSLD	1.4078	Non-Toxin	NON-ALLEGEN
		462	476	VTGLEATLSHRFNEQ	1.2588	Non-Toxin	NON-ALLEGEN
		461	475	KVTGLEATLSHRFNE	1.0888	Non-Toxin	NON-ALLEGEN
	HLA-DRB1*15:01	407	421	NPDLQPEKSRSVEVG	1.4062	Non-Toxin	NON-ALLEGEN
LptD	HLA-DRB1*07:01	489	503	WPILFSTTSSTHILE	0.8205	Non-Toxin	NON-ALLEGEN
		243	257	EYDFRIAGIHQLKPE	1.4081	Non-Toxin	NON-ALLEGEN
	HLA-DRB1*15:01	303	317	TYEIQGYNAQTQVSK	1.2839	Non-Toxin	NON-ALLEGEN
	HLA-DRB1*03:01	451	465	GDAIRVDTNFDPANA	1.0132	Non-Toxin	NON-ALLEGEN

### Molecular docking

**Figure 2:**
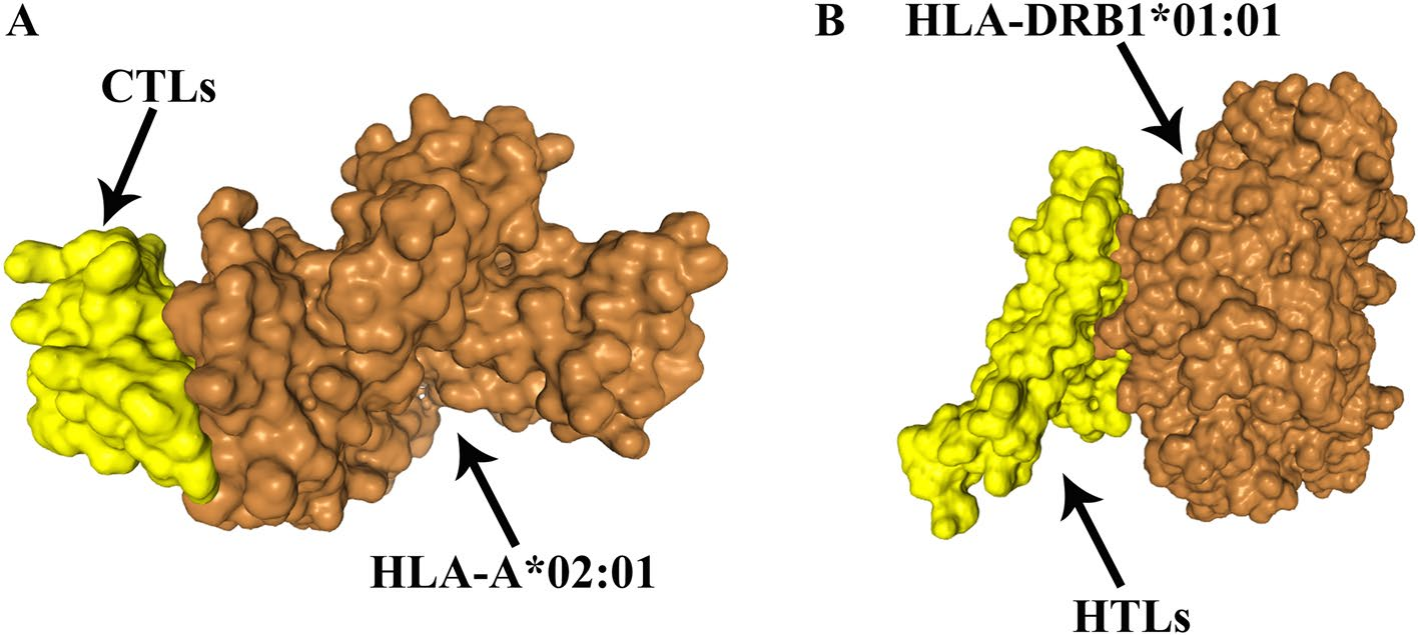
HLA docking with the peptide complexes. (**A**): HLA-A*02:01 (orange) and CTL epitopes (yellow) docking result. (**B**) HLA-DRB1*01:01 (orange) and HTL epitopes (yellow) docking result.

To assess the structural relationship between HLA alleles and T cell epitopes. The HDOCK server provided a list of the top ten docking models, and we chose the first model for evaluation. Molecular docking analysis of CTL epitopes and HLA alleles resulted in a docking score of -251.12 and a ligand RMSD of 48.75 Å (Fig. 2A). The HTL epitopes and HLA allele molecular docking analysis yielded a docking score of -249.48 and a ligand RMSD of 192.84 Å (Fig. 2B).

### Vaccine design

**Figure 3:**
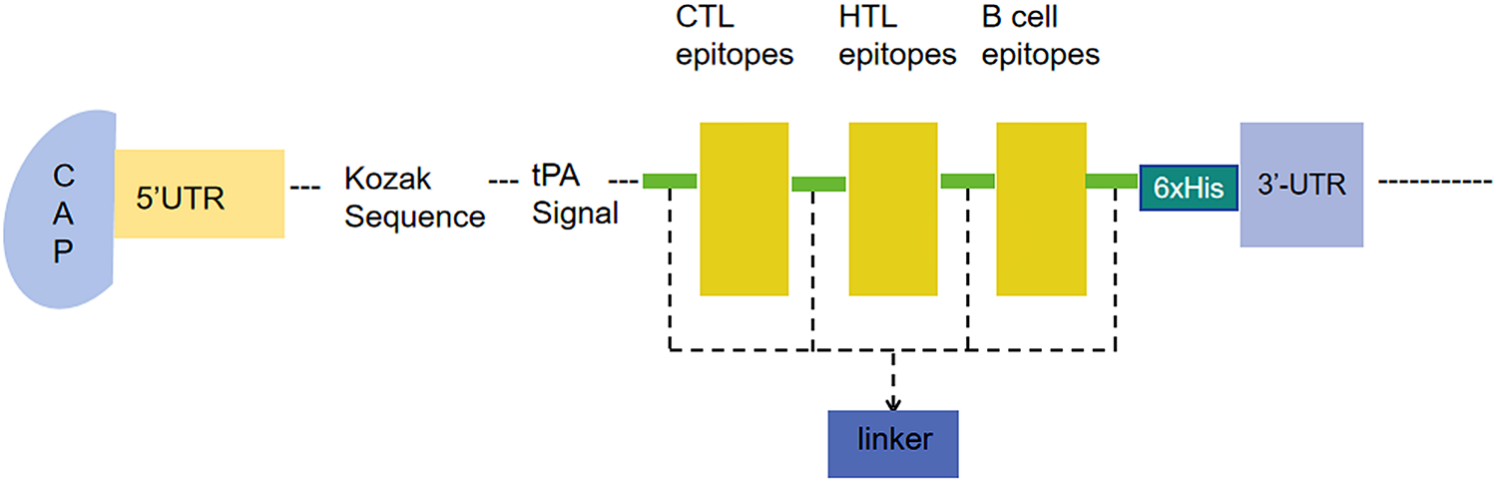
Construct the structural diagram of mRNA vaccine construct from N-terminal to C-terminal. Linkers are AAY, GPPGPG, and KK.

The proposed construction of mRNA vaccine is shown in Fig. 3 MRNA vaccine we designed consists of the following from the N-terminal to C-terminal, that is 5’m7GCap- 5’UTR–Kozak sequence-tPA (Signal peptide)-GPGPGGLNWQATASTSLDMAGPGPGVGLNWQATASTSLDMGPGPGEVGLNWQATASTSLDGPGPGVTGLEATLSHRFNEQGPGPGKVTGLEATLSHRFNEGPGPGNPDLQPEKSRSVEVGGPGPGWPILFSTTSSTHILEGPGPGEYDFRIAGIHQLKPEGPGPGTYEIQGYNAQTQVSKGPGPGGDAIRVDTNFDPANAkkPECIESTNDEPENDENkkQATASTSLDMALYQTRkkMAYIQTRNPGDEKASHkkYSYTMPEPVYGGELNFkkTYEIQGYNAQTQVSKIkkMALRGDAIRVDTNFDPkkPGFSGTNLRFTSEAEWkkYLPSDKYEDEHPNDDSSRAAYFLQGSFNFALAAYLQTYSGISVKAAYNIYMRGMSSKAAYALAPNYDLTLAAYLVYDRDVNTV-GGGSHHHHHH-Stop codon-3’ UTR-Poly (A) tail. (Fig. 3).

### Vaccine physicochemical properties and immunological characterization evaluation

The molecular formula of the vaccine is C_1991_H_3033_N_553_O_635_S_9_. The molecular weight is 45,164.85 kDa, and the number of amino acid residues is 421. The vaccine had a solubility of 0.978002, indicating that the protein antigen was soluble. The instability index (II) was computed to be 28.54, which is less than the threshold of 40, indicating that the vaccine protein was stable. The gravy was − 0.727. The indication shows that the vaccine was a hydrophilic protein. In addition, the vaccine's antigenicity is 1.1082, which is greater than the threshold value of 0.4, indicating that the protein is highly antigenic. According to the allergenicity prediction results, the vaccine is likely non-allergenic. In conclusion, the vaccine design is feasible. Finally, Table 5 presents all the final results.

**Table 5:** The physiochemical profiling of the mRNA vaccine.

Physiochemical profifiling	Measurement	Indication
Number of amino acids	421	Appropriate
Molecular weight	45,164.85	Appropriate
Theoretical pI	5.86	Basic
Formula	C_1991_H_3033_N_553_O_635_S_9_	–
Total number of atoms	6221	–
Total number of negatively charged residues (Asp + Glu)	46	–
Total number of positively charged residues (Arg + Lys)	38	–
Instability index (II)	28.54	Stable
Aliphatic index	55.94	Thermostable
Grand average of hydropathicity (GRAVY)	− 0.727	Hydrophilic
Allergenicity	NON-ALLERGEN	Non-Allergen
Antigenicity	1.1082	Antigenic
Toxicity	Non-Toxic	Non-Toxic
Solubility	0.978002	Soluable

### Simulated immune response

**Figure 4:**
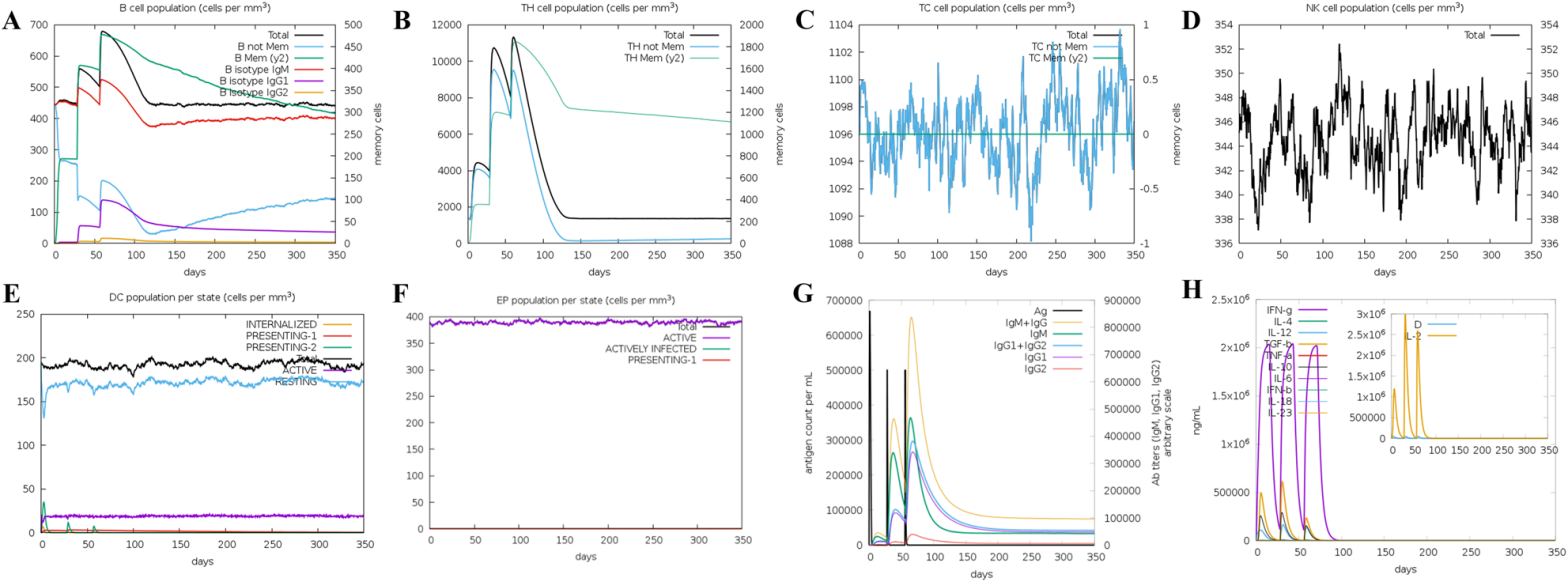
The results of C-ImmSim server immune simulation reaction: (**A**): The B-cell isotypes during each stage. (**B**): The TH isotypes during each stage. (**C**): The TC isotypes during each stage. (**D**): The NK cells during each stage. (**E**): The DC cells during each stage. (**F**): The EP cells during each stage. (**G**): Antibodies of various types during each stage. (**H**): The Cytokines and interleukin during each stage.

After using the C-ImmSim server to simulate the immune response to three vaccine injections, the results showed that the LptD-BTuB mRNA vaccine could induce both cellular and humoral immune responses. B cells were identified to have a part in moderating the immune response, gradually escalating subsequent to each vaccination, and ultimately reaching its zenith (Fig. 4A). Two subsets of T cells, namely TH cells (Fig. 4B) and TC cells, displayed an upward trend and reached a peak following three simulated immune injections. However, the upward trend of TH cells was marginally lower than that of B cells. Contrary to expectations, the levels of TC cells (Fig. 4C), NK cells (Fig. 4D), DC cells (Fig. 4E), and EP (Fig. 4F) remained stable and consistent. Additionally, the data revealed a continuous increase in the concentration of IgM + IgG (Fig. 4G) until it reached its peak. Finally, the vaccine also resulted in alterations of various cytokines (Fig. 4H), particularly IFN and IL-2 cytokines.

### *Codon optimization and *in silico* cloning*

**Figure 5:**
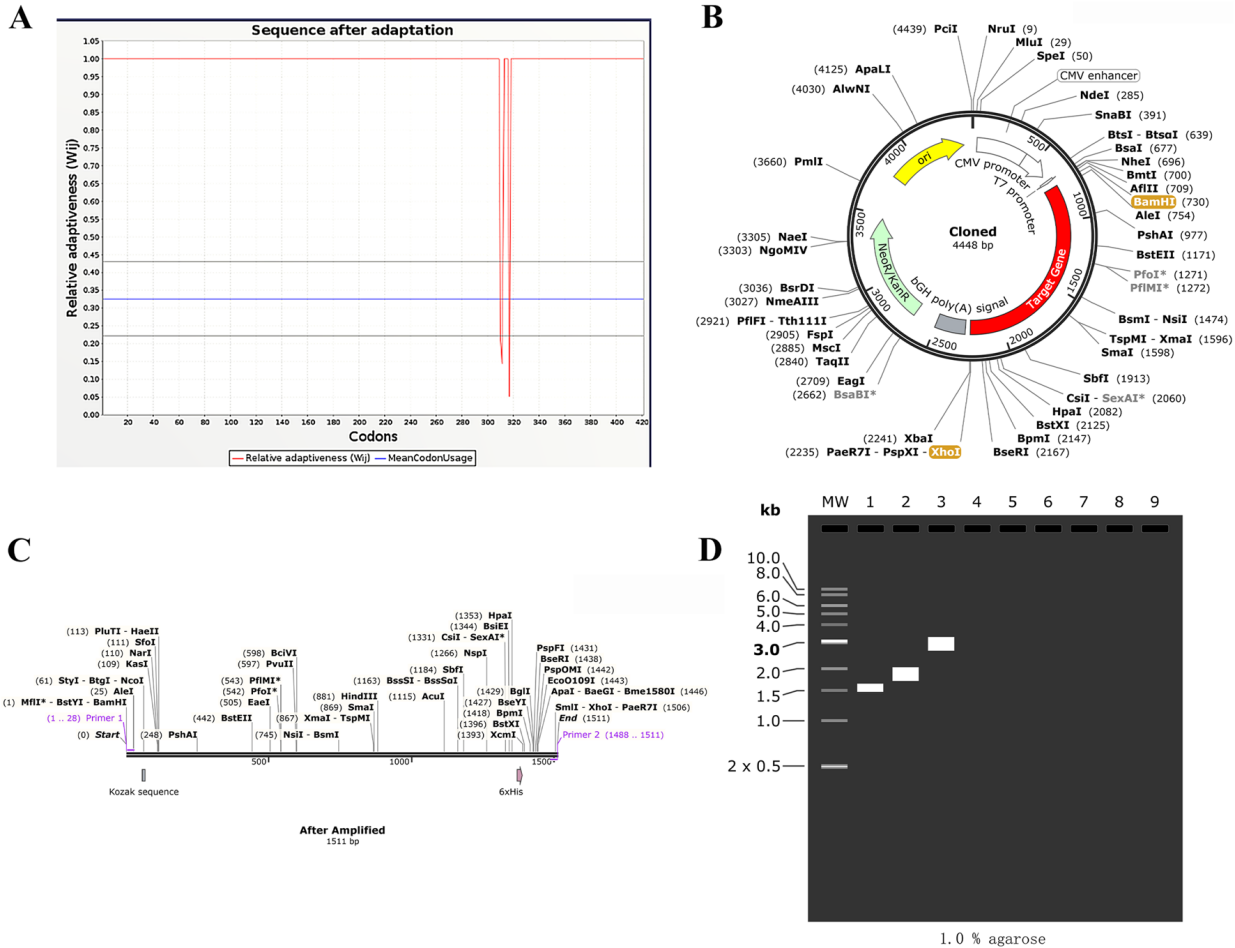
(**A**): Adaptation of optimized codons. (**B**): The constructed recombinant(red) was inserted into the pVAX1 vector(black) in silico cloning. The XHOI and BamHI is restriction endonuclease sites. (**C**): The amplified target gene sequence is 1511bp.D: Simulated agarose gel experiment (1.0% agarose ). As shown in the figure, “1” stands for LptD-BtuB mRNA vaccine, “2” stands for pVAX1, and “3” stands for recombinant illustrated in the fgure, “1” stands for the MEV, “2” stands for pET-28a ( +), and “3” stands for recombinant plasmid.

The amino acid sequence of the fused epitope protein was successfully converted to nucleotide sequence using the online server EMBOSS Backtranseq. The nucleotide sequence was then optimized with the JCat tool, excluding XHOI and BamHI restriction endonuclease sites. E. coli served as the host for the optimization process. Finally, the optimized codon adaptation index (CAI) value is 0.98, with an average percentage of GC content percentage of 53%(Fig. 5A).The 5' and 3' ends of the target gene were modified with BamHI and XhoI restriction sites. Design the primers with the following specifications: The forward primer sequence is 5’GGATCCACATTTGCTTCTGACACAACTG, with a length of mer, a Tm value of 62°C, and GC content of 46%. The downstream primer sequence is 5’CTCGAGTGCTGCCCACTCAGACTT, with a length of 24-mer, a Tm value of 64°C, and GC content of 58%.The target gene was amplified using simulated PCR in SnapGene and inserted into the multiple cloning site (MCS) region of the plasmid after removing the previously designed primer restriction site. The plasmid sequence of pVAX1 is 2999 bp, while that of the recombinant plasmid is 4448 bp (Fig. 5B) The target gene sequence is 1511 bp (Fig. 5C). Finally, a 1% agarose was selected for the simulated electrophoresis reaction (Fig. 5D) (see Supplementary Fig. 1).

### mRNA Vaccine’s and peptides’s structure

**Figure 6:**
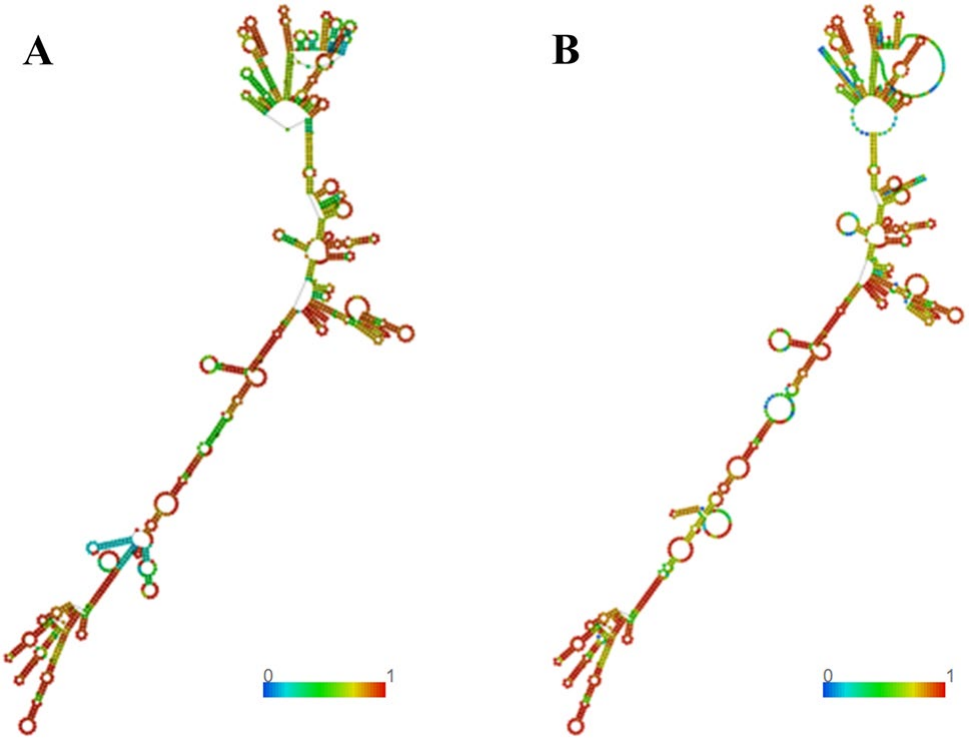
Prediction of secondary structure of mRNA using RNAfold website. (**A**): Optimal secondary structure: the minimum corresponding free energy. (**B**): Centroid secondary structure.

RNAfold predicts the RNA's secondary structure, producing two structures as output, which are the minimum corresponding free energy (MFE) and the centroid structure. The centroid structure that contains a minimal base-pair distance to all structures in the thermodynamic ensemble. After inputting the optimized vaccine codon sequence, the optimal secondary structure is obtained, guaranteeing the minimum corresponding free energy (MFE) of − 495.13 kcal/mop (Fig. 6A). The other is a secondary centroid structure with a stability value of − 401.50 kcal/mol (Fig. 6B). The smaller value indicates greater stability. Predicting the secondary structure of mRNA is significant in enhancing its stability to withstand endonuclease cleavage and chemical degradation.

**Figure 7:**
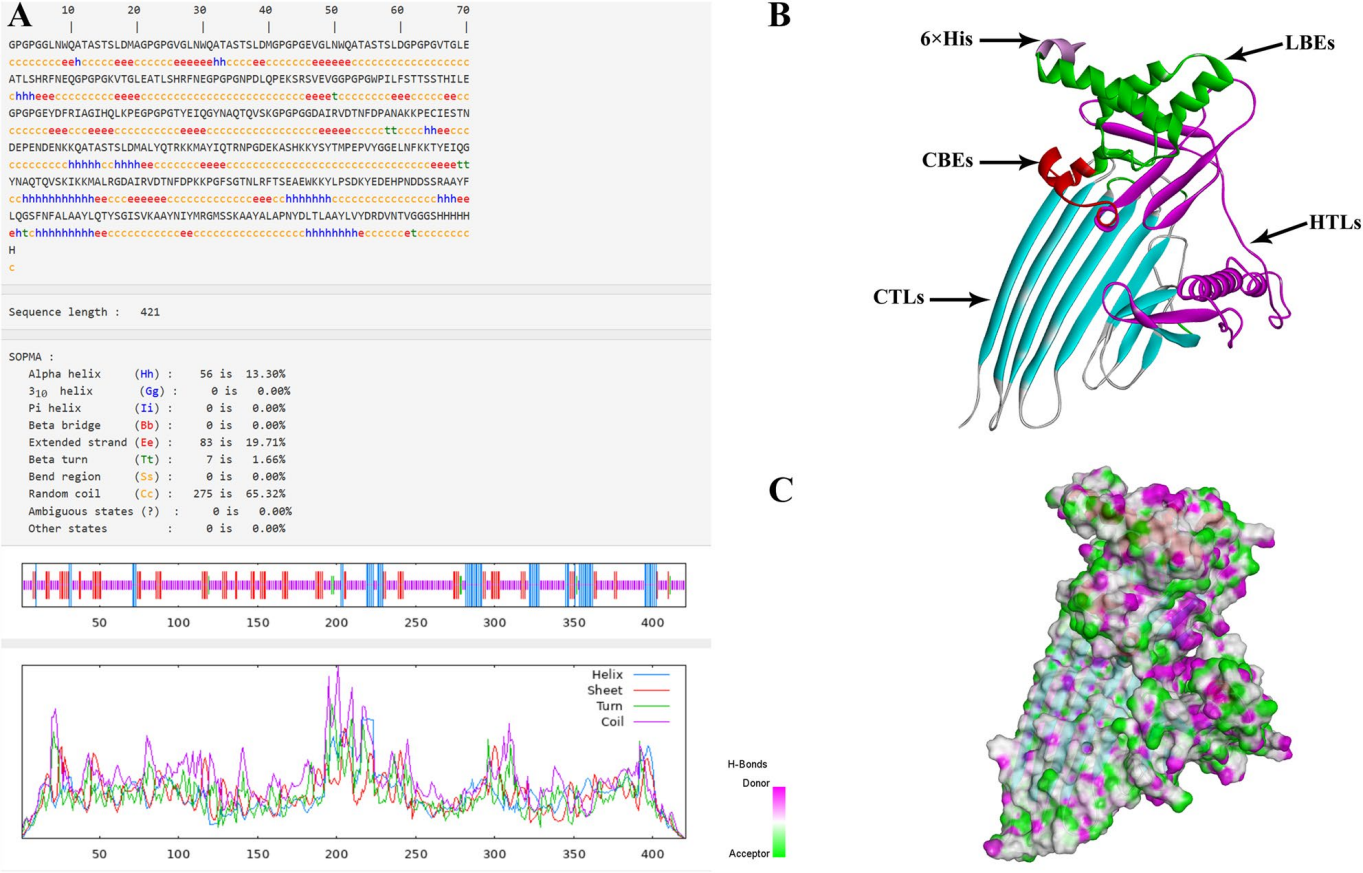
(**A**) :The Secondary structure of LptD-BTuB mRNA Vaccine. (**B**): The tertiary structure LptD-BTuB mRNA Vaccine. (**C**) :H-Bonds of vaccine. As shown in the figure, pink areas: donors, green areas: recipients.

**Figure 8:**
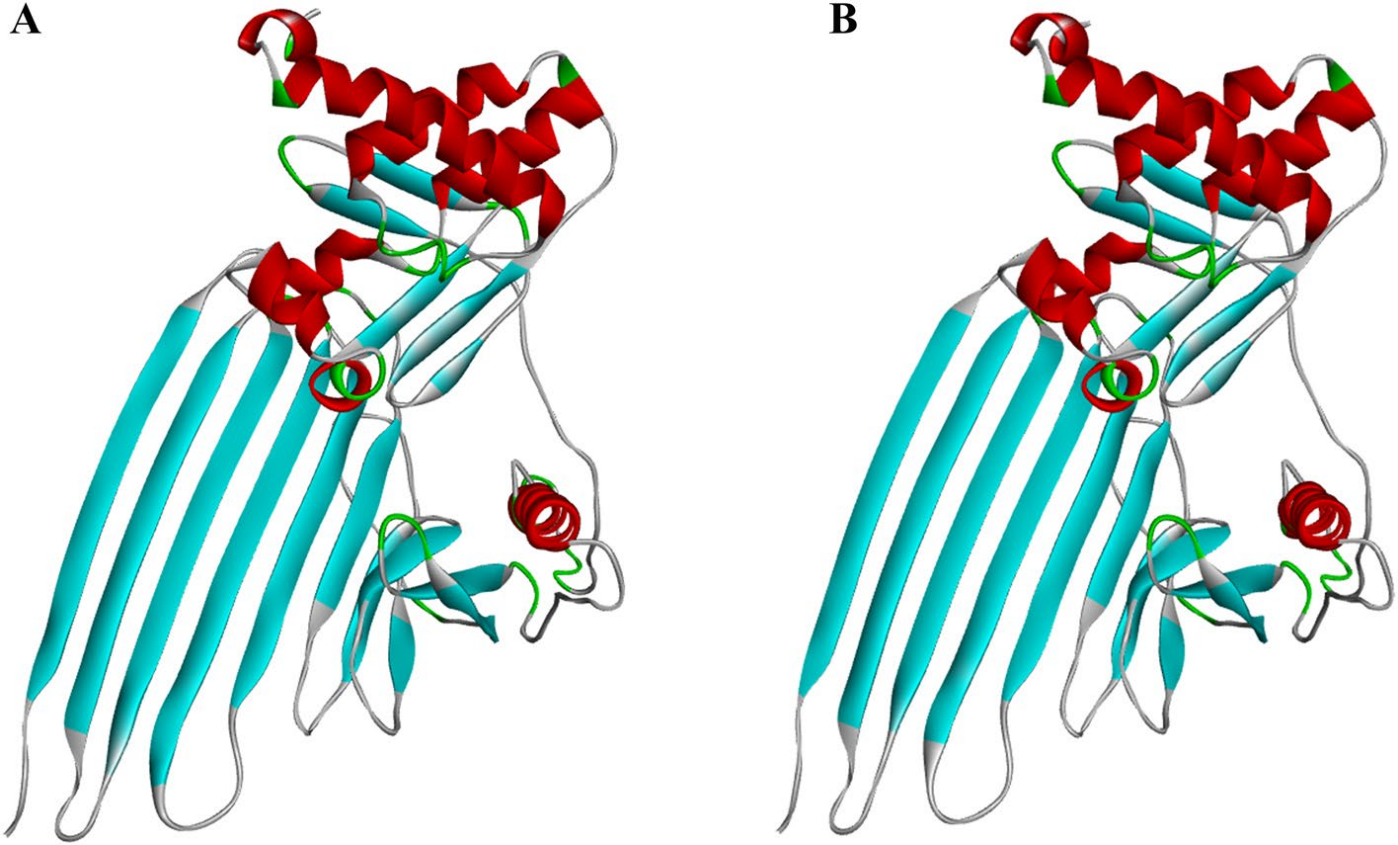
(**A**) :The unoptimized tertiary structure of LptD-BTuB mRNA Vaccine. (**B**)**:** Optimized the three-level structure of GalaxyRefine.

The peptide's two-dimensional structure, as predicted by SOPMA, is depicted in Fig. 7A, consisting of 13.30% alpha-helix, 1.66% β-turn, 65.32% random coil, and 19.71% extended strand. The ratio of The alpha-helix, random coil, extended strand andβ-turn the secondary structure aligns with that of the tertiary structure. This suggests that the tertiary structure (Fig. 7B) predicted by Robetta is accurate. We selected model one out of the five models, and its confidence level is 0.40. The three-level structure was optimized using GalaxyRefine (Fig. 8). Next, we used Discover Studio to display the PDB format of the vaccine. The pink areas in the model represent the donor, while the green areas represent the recipient (Fig. 7C). Following up with an analysis and application of the optimized three-level structure model.

### Evaluation of tertiary structure quality

**Figure 9:**
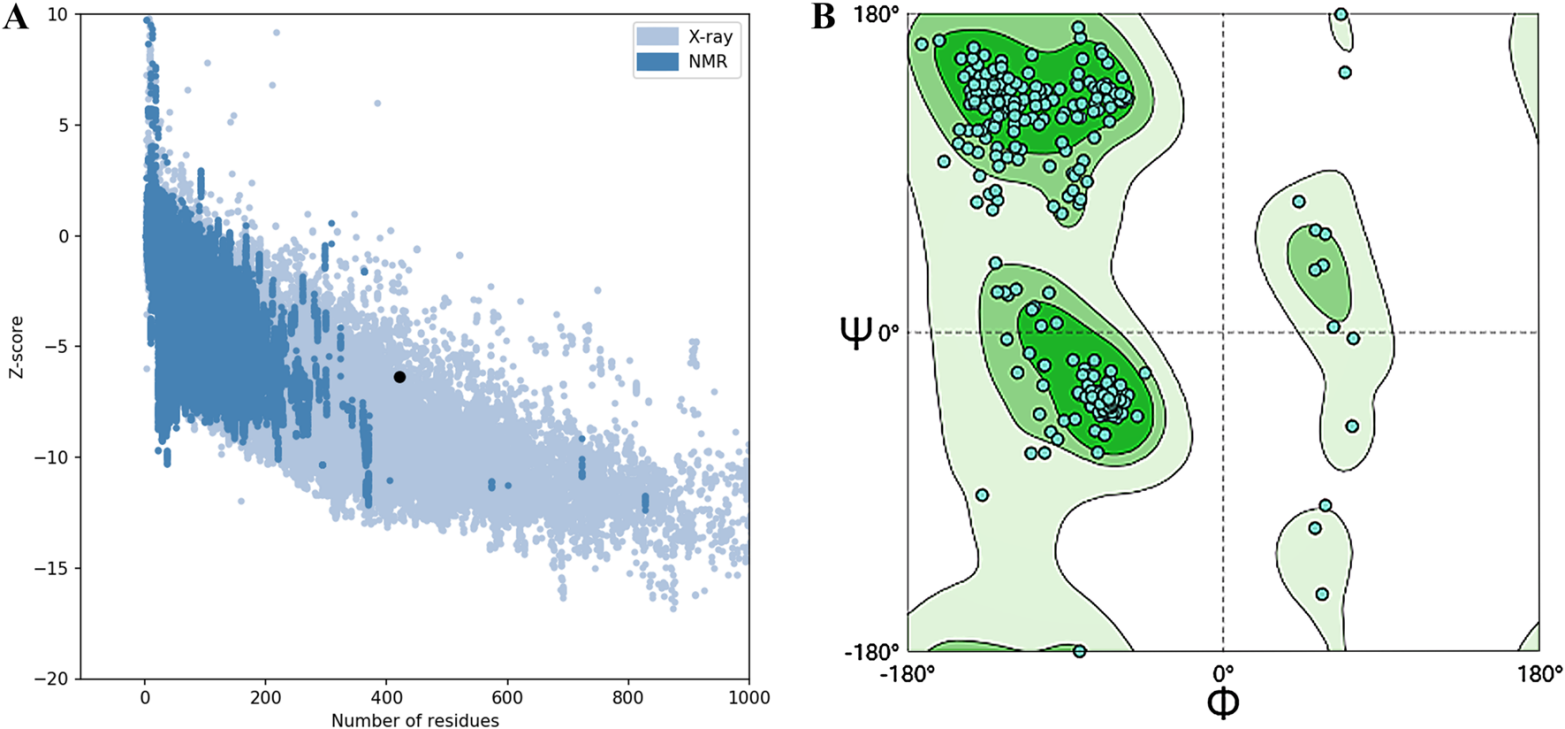
(**A**): The Z-score plot is obtained from the ProSA-web. (**B**): Validation: Ramachandran plot analysis showing 94.75% in favored, 5.15% in allowed, and 0.72% in disallowed regions of protein residues.

Using ProSA-web server to evaluate the predicted the model of tertiary structure (Fig. 9A), The light blue region in the figure represents the source of X-rays in the predicted tertiary structure, and the dark blue region represents the source of nuclear magnetic resonance (NMR) in the predicted tertiary structure. Verification of the model was performed with the ERRAT quality factor of 80.110 (see Supplementary Fig. 2 )while Verify 3D score was 92.40%(see Supplementary Fig. 3 ). The Z-score of the LptD-BTuB mRNA vaccine was -6.36, indicating the protein has the correct structure. In order to ensure the accuracy of structural prediction again, we use SWISS-MODEL online service and predicted using the Ramachandran plots. The allowable area as the dark green region and restricts the maximum allowable area to the light green region (Fig. 9B). The blank area is prohibited. Ramachandran plot analysis showing 94.75% in favored, 5.15% in allowed, and 0.72% in disallowed regions of protein residues. Based on the figure, a significant number of amino acids fall within the specified range, leading to a conclusion that the three-level structure prediction model for the LptD-BTuB mRNA vaccine is highly credible.

### Molecular docking

**Figure 10:**
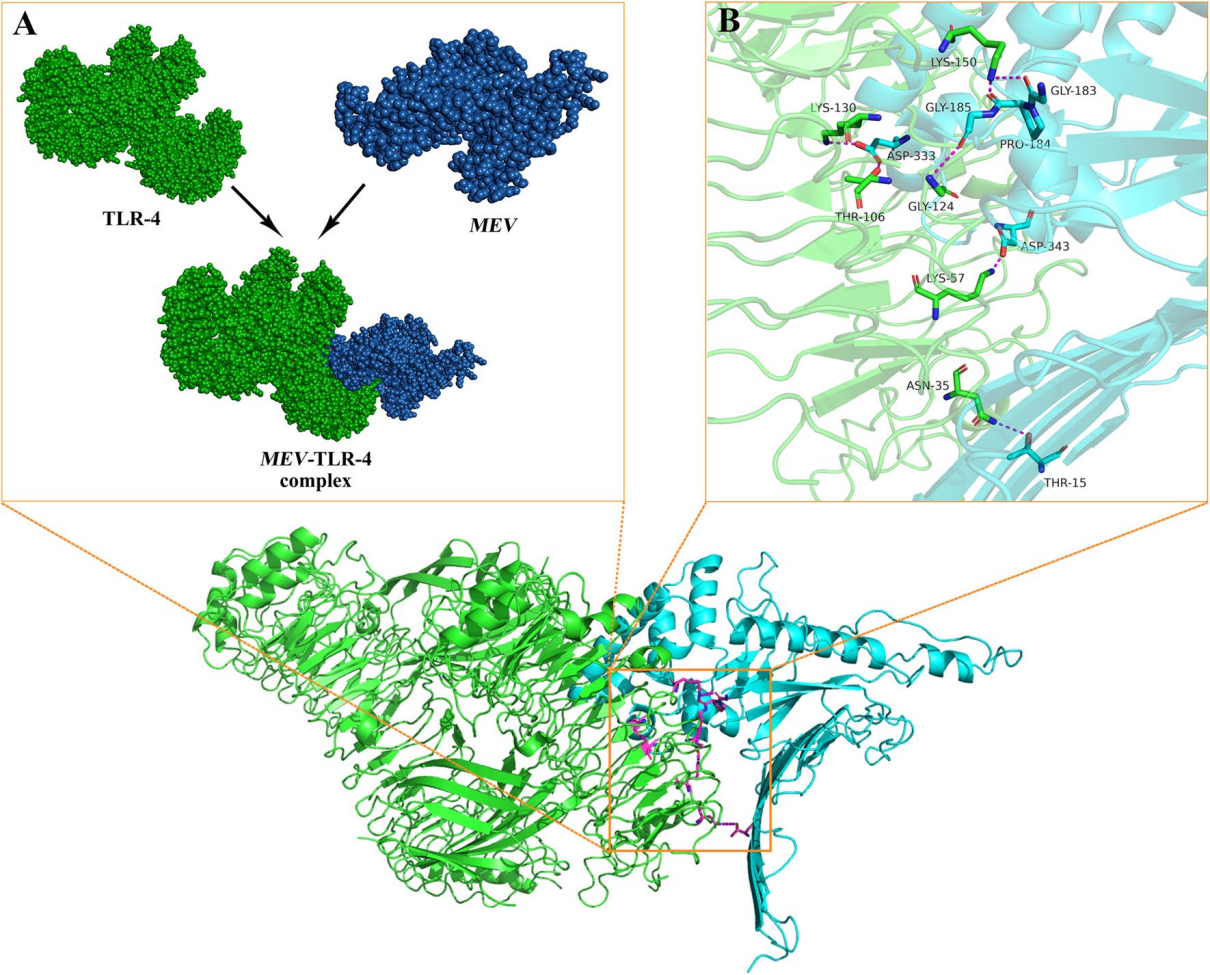
The vaccine is docked with TLR4. (**A**): Vaccine -TLR-4 docking complex. (**B**): Vaccine -TLR-4 complex was analyzed for interactions using PyMol three-dimensional visualization.

The HDOCK website utilizes a semi-flexible docking approach while taking into account non-bonding interactions, including electrostatic interaction and van der Waals force, to forecast its binding mode and affinity. The website provided a list of the top ten docking models, and we selected the first model for evaluation. The results of the molecular docking analysis indicate a docking score of − 364.59, a ligand RMSD of 93.69 Å and a confidence score of 0.9865 in Fig. 10A.We utilized PyMOL for three-dimensional visualization. The purple dotted line in the Fig. 10B represents hydrogen bonding.

### Molecular dynamic simulation

**Figure 11:**
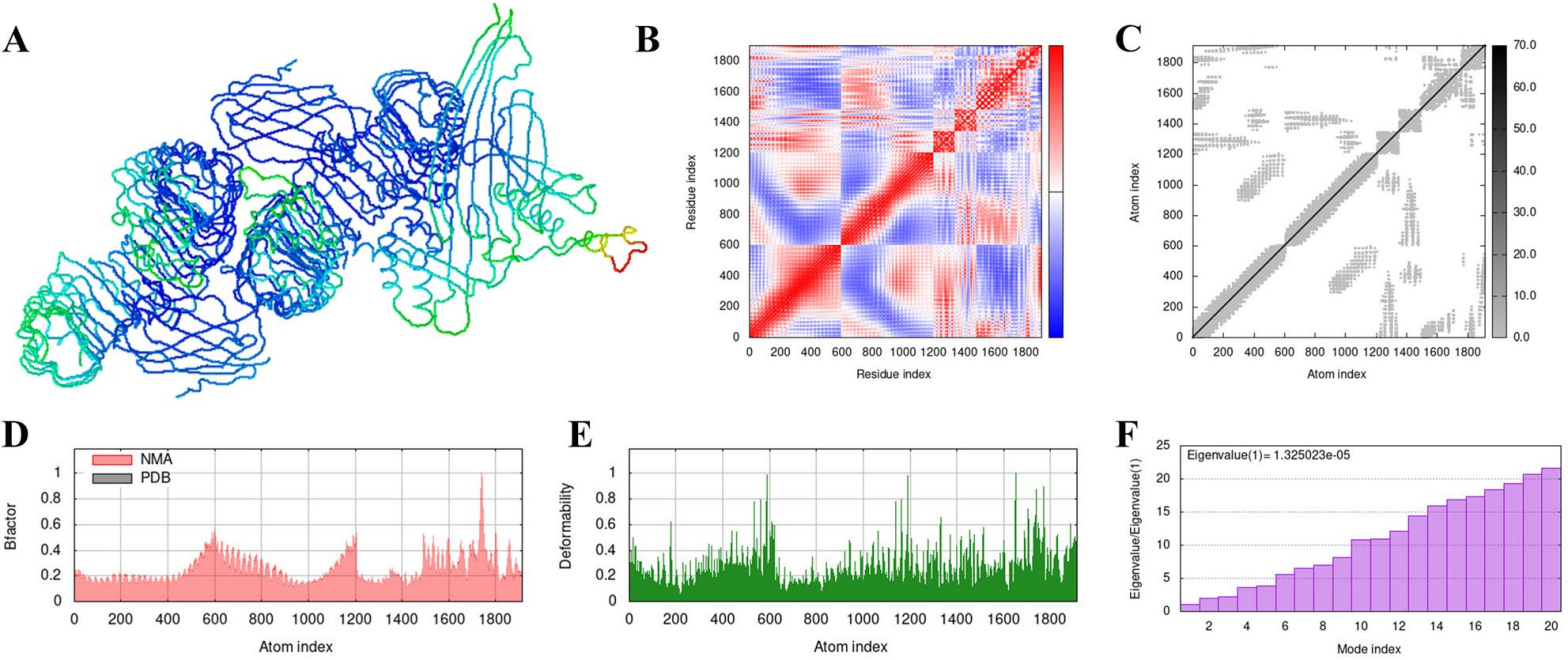
The receptor–ligand interactions. (**A**): The docking of mRNA vaccine and TLR4 using the Cluspro wedsite. (**B**): Covariance matrix. (**C**): elastic network model. (**D**): B-factor graph. (**E**): deformability graph. (**F**) :Eigenvalue of the vaccine–TLR4 complex.

The IMOD server conducts molecular dynamics simulations of TLR4 complexes under specific conditions. The binding of mRNA vaccine and TLR4 is shown in Fig. 11A. A service provided by Cluspro. Within the dynamic region, the covariance matrix illustrates the relationship between amino acid dimers in Fig. 11B. The red section represents related residues, the white denotes anti-related residues, and the blue indicates irrelevant residues. In Figs. 11C, the elastic network model is represented by ordered pairs of atoms connected by springs, with the gray area in the figure indicating the hard region. Therefore, the constructed vaccine constructed can be considered to be harder and more stable. The B-factor graph illustrates the correlation between the Normal Mode Analysis and PDB regions of the complex in Fig. 11D. The flexible regions of the structure represent amino acids with high deformability, as reflected in the peak map of deformability shown in Figs. 11E.The eigenvalues of the two docking complexes are shown in the Fig. 11F.

## Discussion

*Brucella*, a Gram-negative coccidian, has the potential to cause zoonotic diseases and lead to public health incidents, economic losses, and casualties worldwide. Therefore, it is crucial to understand and prevent the spread of this pathogen^1^. For humans, the most pathogenic species are *Brucella melitensis, Brucella abortus* and *Brucella suis*. Brucellosis is potentially harmful with variable clinical manifestations , and no effective treatment is available ^24^. Vaccination remains the most effective preventive measure^25^. Development of highly effective vaccines against human *Brucella* remains a public health priority^26^. In our study, we designed the multi-epitope mRNA vaccine, which provided novel strategies for developing the vaccine.

The primary attenuated live vaccines for brucellosis in China include *Brucella melitensis* strain M5, *Brucella suis* strain S2, and *Brucella abortus* strain A19^27^. However, there are many shortcomings, such as persistent infection in vaccinated animals, risk of toxicity reversal, lack of lasting protection, interference with brucellosis diagnosis and so on^28^.Developing a new vaccine needs to verify its effectiveness and safety, which is a time-consuming and expensive process. Therefore, using immunoinformatics approaches to design new kind of vaccines could be a magnificently additive in the way forward of preventing *Brucella*. Many studies focused on design of novel vaccines using bioinformatics tools^29^. Subunit vaccines are developed utilizing fragments of pathogens like proteins^30^. Subunit vaccines encompass a range of types such as include recombinant protein vaccine, epitope vaccine, DNA vaccine, gene marker vaccine, conjugate vaccine, recombinant vector vaccine, nanoparticle vaccine, bacterial shadow vaccine, mRNA vaccine and so on^31^. Compared to other vaccines, mRNA vaccines have obvious advantages. First, they are safe, as there is no potential risk of infection or insertion of mutant genes, and mRNA is degraded through normal cell processes. Secondly, using various modifications can make mRNA more stable and highly translatable. Thirdly, the in vitro transcription reaction yields a high amount of mRNA, which gives mRNA vaccines the potential for rapid, cost-effective, and large-scale production^32^.

A qualified vaccine should also contain effective epitopes of cytotoxic T lymphocytes (CTL), helper T lymphocytes (HTL), and B cells^33^, thus has the capability to induce the activation of both cellular and humoral immune responses. In this study, the epitopes of the LptD and BtuB proteins were analyzed and predicted. Before making the prediction, we removed the signal peptide from the protein. The signal peptide can impact the initial stage of protein translation and even affect the subsequent folding and transportation of the protein^34^. In this study, we found 5 CTL epitopes and 10 HTL epitopes using IEDB NetCTLpan1 and NetMHC-IIpan-4.0. T cell epitopes and HLA molecules are well matched. The incidence of brucellosis in Xinjiang is much higher than that of most provinces in China^35^. Therefore, our focus is on the high-frequency alleles of the Xinjiang population to predict T cell epitopes. We selected the alleles with high frequency in Xinjiang (HLA-A*1101, HLA-A*0201, HLA-A*0301, HLA-DRB1*0701, HLA-DRB1*1501, and HLA-DRB1*0301) to predict the cytotoxic T lymphocyte (CTL) epitopes and helper T lymphocyte (HTL) epitopes.T-cell epitopes must be processed by antigen-presenting cells and recognized by T-cell receptors. When antigen-presenting cells (APC) present antigen peptides to T cells, they are assisted by both MHC I and MHC II molecules. Cytotoxic T lymphocytes (CTL) recognize MHC I, while helper T lymphocytes (HTL) recognize MHC II. In this study, we predicted 8 B cell epitopes using IEDB and ABCpred. In order to optimize immunogenicity, 8 B cell epitopes and 15 T cell epitope were combined with flexible lingers, to produce a novel vaccine. The B-cell epitopes and T-cell epitopes added in vaccine help to enhance the humoral and cellular immune response^36^.

In our study, several unique elements are included in mRNA vaccines for improving mRNA stability and translation efficiency. The 5' and 3' UTR elements on both sides of the coding sequence can originate from viruses or eukaryotic genes^37^. This significantly impacts the stability and translation of mRNA, leading to a substantial increase in mRNA half-life and expression^38^. A 5' cap structure is necessary for the effective translation of mRNA into protein^39^. The poly(A) tail plays an important role in mRNA translation and stability, and can be directly added to mRNA using poly(A) polymerase^40^. In ribosome translation, protein synthesis will stop when it encounters the conservative stop codons: UAA, UAG, or UGA^41^. Kozak sequence is recognized by ribosomes as translation initiation site^42^. TPA^43^ is a sequence located before the coding region in a structural gene, which can be transcribed but not translated. The open reading frame is the translation area of mRNA, and replacing rare codons with common codons is beneficial to the efficiency of subsequent translation^44^. We added a 6xHis tag to the C-terminal of the fused amino acid sequence to facilitate protein purification through affinity chromatography^45^.

An adjuvant is a substance that can nonspecifically enhance or alter the specific immune response of the body to the corresponding antigen, increase the antigen's immunogenicity, or modify the type of immune response, but does not have antigenicity itself^46^. Adjuvants in mRNA vaccines are typically categorized into three groups: (1) RNA with inherent adjuvant activity from its nucleotide sequence or encoded protein; (2) components of the delivery system, particularly ionizable cationic lipids in lipid nanoparticles^47^; and (3) exogenous immunostimulants^48^. Lipid nanoparticles (LNPs) have become one of the most popular and widely used tools for delivering mRNA^49^. LNP typically consists of four components: ionizable cationic lipids, which facilitate self-assembly into virus-sized particles (approximately 100 nm) and enable the release of mRNA endosomes into the cytoplasm; Lipid-linked polyethylene glycol (PEG), which prolongs the preparation's half-life; Cholesterol as a stabilizer; and naturally occurring phospholipids that maintain the lipid bilayer structure^32^. In this study, the adjuvant is not directly connected to the vaccine, because mRNA vaccines often are supplemented with lipid nanoparticles, serving as both a carrier vector and an adjuvant, boosting the immune response.

One of the key issues in vaccine is the capacity to elicit the desired immune response and generate immune factors following injection into the body. In our study, the Online website C-ImmSim was employed to perform Immune Simulation Response.After simulating the three-needle injection reaction, B cells can differentiate into plasma cells when stimulated by antigens, and plasma cells can produce and release antibodies. After the simulated injection, there is an increase and peak in the amount of B cells, along with elevated levels of antibody IgM and IgG. This demonstrates the production of an effective humoral immunity. The rise in the quantity of natural killer (NK) cells and dendritic cells (DC) suggests that vaccination typically generates innate immunity. Additionally, it can trigger alterations such as the presence of IFN and IL-2 cytokines.

## Conclusion

The physical and chemical characteristics of the LptD-BTuB mRNA immunization formulated in this study fulfill the fundamental criteria for a vaccine. After simulating immunity with the C-ImmSim server, it has been theoretically proven that the vaccine can result in optimal cellular and humoral immunity post-injection. However, to ascertain the practical utility of the vaccine in brucellosis prevention, further experiments are crucial, despite challenges that would be expected during the experimental process.

## Materials and methods

### Obtaining protein sequences

Epitopes refer to specific regions on the surface of antigens that are identified by the immune system^50^. In this study, the conserved region of the chosen protein was retrieved from the National Center for Biotechnology Information (NCBI)^51^ (https:// www.ncbi.nlm.nih.gov/accessed) and were (1) LPS assembly protein LptD (accession number WP_011068938.1) lptD, (2)TonB-dependent receptor Outer membrane BTuB(accession number WP_004691650.1 )1), which were identified as potential protective antigens^19^.

### Immunological characterization

As antigenicity is a crucial factor in vaccine development, we initially predicted antigenicity. As antigenicity is a crucial factor in vaccine development, we initially predicted the antigenicity^52^. The ANTIGENpro (https://scratch.proteomics.ics.uci.edu/) website was conducted to confirm the antigenicity of the selected protein,which is a part of Scratch protein predicter. The physicochemical properties of the protein were analyzed using the online software ProtParam (http://web.expasy.org/protparam/), including molecules the number of amino acids, the molecular formula, molecular weight, the instability index, and the overall mean of the water solubility (GRAVY). In order to conduct a more comprehensive analysis of the selected proteins.

AlgPred (https://webs.iiitd.edu.in/raghava/algpred/submission.html) predicts the allergenicity of bacterial protein sequences which choose prediction a MEME/MAST motif aproach. Using the online website Gneg-mPLoc (http://www.csbio.sjtu.edu.cn/bioinf/Gneg-multi/ )to predict the subcellular localization of two quantitative proteins again.

### Prediction of signal peptides

When analyzing the structure of a protein and predicting the epitope, the signal peptide sequence should be removed first^53^.To predict the presence of a signal peptide in the selected protein, we uesd SignalP 5.0^54^ and LiPOP 1.0 software (https://services.healthtech.dtu.dk/service.php?LipoP-1.0) to analyze the protein sequence for potential signal peptides. SignalP 5.0 can predict signal peptides (SPs) and their associated cleavage sites in proteins from Gram-negative bacteria. Meanwhile, LipoP 1.0 functions as a signal peptide (SP) prediction tool that differentiates between lipoprotein SPs, other SPs, and N-terminal membrane helices in Gram-negative bacteria^55^. We will combine the two results to remove the signal peptide.

#### T cell epitopes prediction

T cells can only recognize antigen-presenting cells (APCs) through their interaction with major histocompatibility complex (MHC) molecules, rather than directly identifying epitopes^56^. MHC molecules are primarily classified into two categories: class I (MHC I) and class II (MHC II)^57^. Cytotoxic T lymphocyte (CTL) cells recognize endogenous antigenic peptides presented by MHC I molecules, while helper T lymphocyte (HTL) cells recognize exogenous antigenic peptides presented by MHC II molecules^58^. Therefore, we found it necessary to analyze the CD8 + and CD4 + T cell epitopes separately. For this study, we chose to use the high-frequency alleles of Xinjiang, which consist of HLA-A*1101 (13.46%), HLA-A*0201 (12.50%), HLA-A*0301 (10.10%), HLA-DRB1*0701 (16.35%), HLA-DRB1*1501 (8.65%), and HLA-DRB1*0301 (7.69%), to predict the CTL and HTL epitopes^59^.

The CTL epitopes were predicted using IEDB (http://tools.immuneepitope.org/) and the NetCTLpan1 server (https://services.healthtech.dtu.dk/service.php?NetCTLpan-1.1). Amino acids with a length of 10 mers were selected based on the previously mentioned alleles, with no modification to the original parameters. The top ten epitopes with the highest scores from the software were chosen for further investigation. HTL epitopes of target proteins will be predicted using the IEDB and NetMHC-IIpan-4.0 (https://services.healthtech.dtu.dk/service.php?NetMHCIIpan-4.0), with an amino acid length of 15 mer. The default thresholds for NetMHC-IIpan-4.0 will remain unchanged. After running the predictions on both servers, the top ten epitopes will be selected for further analysis.

#### B cell epitopes Prediction

Antigens use B cell epitopes to recognize B cells and stimulate the B cell immune response^60^. We decided to use Ellipro from IEDB (http://tools.iedb.org/ellipro/) and ABCpred (https://webs.iiitd.edu.in/raghava/abcpred/ABC_submission.html) to identify B-cell epitopes. The length of the epitopes was set at 16 mer. We will select the top ten epitopes from the epitope prediction results provided by the online servers, and screen for cross-reactive epitopes for subsequent verification.

### Homology of predicted peptides with humans

Non-homologous proteins can reduce the likelihood of stimulating the body to produce an immune response^61^. By using the NCBI BlastP database (https://blast.ncbi.nlm.nih.gov/Blast.cgi?PAGE=Proteins), a comparison was made between Homo sapiens (TaxID: 9606) and predicted peptides to mitigate the risk of autoimmunity. Peptides with an E value greater than 0.05 were considered non-homologous and suitable for use in vaccine development^62^.

### Antigenicity, allergenicity and toxicity evaluation of epitopes

All selected epitopes need to be evaluated for their antigenicity, allergenicity, and toxicity^63^. To assess antigenicity, the VaxiJen online web server (http://www.ddg-pharmfac.net/Vaxijen/VaxiJen/VaxiJen.html ) was performed with the bacteria parameter and a threshold of 0.4 were selected. Using the website of AllerTop V.2.0 (http://www.ddg-pharmfac.net/AllerTOP) to analyze the allergenicity of epitopes, which predicts using a Tanimoto coefficient to predict^64^. The ToxinPred server was utilized to evaluate the toxicity of the selected epitopes. Ultimately, only epitopes that were antigenic, non-toxic, and non-allergenic were chosen for further analysis.

### Molecular docking of T-cell epitopes to HLA alleles

The human leukocyte antigen (HLA) system is an integral component of the human immune system. The trait is controlled by genes located on chromosome 6 and encodes cell surface molecules. These molecules present antigen peptides to T cell receptors (TCR) on T cells^65^. In order to maximize vaccine coverage within the population, it is important to consider the frequency of HLA alleles^66^. The HLA DRB*0101 allele is associated with susceptibility and is present in 95% of the population^67^. HLA class I (HLA-A*02:01) is one of the most common HLA in the world^68^. The HDOCK server^69^ was utilized to molecular docking, following selection of HLA class I (HLA-A*02:01) and HLA class II (HLA-DRB1*01:01) alleles were chosen for molecular docking with T cell epitopes. The resulting interactions between the alleles and T cell epitopes were subsequently identified.

### Construction of mRNA vaccine

The construction structure of mRNA vaccine from N-terminus to C-terminus includes the following components:5’m7GCap-5’UTR-Kozak sequence-tPA (Signal peptide)-GPGPG (linker)-HTL Epitopes-KK (linker)-B Cell Epitopes-AAY (Linker)-CTL Epitopes-GGGS (linker)-HHHHHH-Stop codon-3’UTR-Poly (A) tail. We have given this vaccine the name LptD-BTuB mRNA vaccine.

GPGPG^70^, KK, AAY^71^ and GGGS^72^ linkers were used to connect all of the predicted epitopes. The linkers' purpose is to separate epitopes, ensuring that their functions—flexibility, cuttability, and solidity—are not interfered with. The KK linker can maintain the independent immune activity of B-cell epitopes in vaccines^73^. AAY linker can improve the immunogenicity of an epitope vaccine^74^. The GPGPG linker will facilitate the immune processing and presentation processes^75^.To detect and purify recombinant proteins later on, we plan to add a group of 6xHis tag sequences to the C-terminal of the amino acid sequences mentioned above ^76^. MRNA vaccine^17^ must be have a Kozak sequence that contains a start codon AUG, at the beginning of ORF and a stop codon TGA ended translation. Moreover, This construction contains three structures. Firstly, the leader sequence of tissue plasminogen activator (tPA) (UniProt ID: P00750) is located in the 5 regions of the construct, which ensures correct translation of the target gene fragment. Secondly, the 5' and 3' Untranslated regions (UTRs) will consist of human β globin (NCBI Gene ID: 3043) and α globin (NCBI Gene ID: 3039), respectively.

#### Physicochemical properties, antigenicity, allergenicity, and toxicity of vaccine

Using the VaxiJen^77^ and ANTIGENpro(http://scratch.Proteomics.ics.uci.edu/) servers to perform the assessment of antigenicity of the constructed mRNA vaccines, which only the amino acid sequence was included, without the tPA or MITD sequences. The allergy potential of the construct was assessed using the AllerTOP 2.0 server, while the ToxinPred server was utilized to evaluate its toxicity^78^. Physicochemical parameters of the construct, such as amino acid concentration, theoretical isoelectric point (PI), molecular weight, Aliphatic Index (AI), Instability Index (II), and Grand Average of Hydropathicity (GRAVY), were estimated using the program website ExPASy-ProtParam(https://web.expasy.org/protparam/)^79^. And the SOLpro (http://scratch.proteomics.ics.uci.edu/) was applied to predict the solubility^80^.

### Immune simulation response

The Online website C-ImmSim simulation (http://150.146.2.1/C-IMMSIM/index.php) was used to evaluate the efficacy of immunological simulation with the vaccine. Its mechanism is as follows, It can simulate the immune response of epitope interacting with T cell receptor. We have chosen to analyze high frequency alleles in Xinjiang, specifically HLA-A*1101, HLA-A*0201, HLA-B*5101, HLA-B*3501, HLA-DRB1*0701, and HLA-DRB1*1501.The server is capable of simulating three components of mammalian immune system: bone marrow, thymus, and lymph nodes. The intervals between the three vaccine injections were 1, 84 and 168 time-steps respectively. 1 time-steps equivalence of 8 h of real-life, the three vaccine injections was administered at intervals of 0, 28, and 56 days^67^. Each injection contains a dose of 1000 units^75^.Ultimately, the simulation parameters were set as follows: random seed 12,345, simulation volume of 50, and simulation step of 1050.

#### Optimization of Codon of mRNA Vaccine and in silico cloning

Firstly, step one was transform amino acid sequence into a nucleotide sequence ,which would use the online server EMBOSS Backtranseq(https://www.ebi.ac.uk/Tools/st/emboss_backtranseq/) to complete. The criteria for optimization were a Codon Adaptation Index (CAI) greater than 0.8 and a GC content between 40–70%. In the second step of the process, we utilized the JCat tool (http://www.jcat.de/Literature.jsp) to optimize the codon usage for the mRNA vaccine. We also eliminated the XHOI and BamHI restriction endonuclease sites. These efforts resulted in the acquisition of the LptD-BTuB mRNA vaccine DNA sequence according to the generated output. The next step in the in silico cloning process was to select pVAX1 as the vector. SnapGene software (https://www.snapgene.com/) was then utilized to design primers and conduct a polymerase chain reaction (PCR) with primer lengths ranging from 15 to 30 base pairs, Tm values set at 60°C, an annealing temperature of 1°C, and GC content between 40 and 60%. Finally, the LptD-BTuB mRNA vaccine DNA fragments will be inserted into the cloned plasmid using the two restriction sites that were selected.

#### Structure prediction for mRNA vaccine and peptides

The ViennaRNA Package 2.0's RNAfold tool (http://rna.tbi.univie.ac.at/cgi-bin/RNAWebSuite/RNAfold.cgi) was utilized to predict the secondary structure of the mRNA vaccine we created. The McCaskill method was used to calculate the minimum free energy (MFE) of the expected secondary structure.

In order to determine the secondary and tertiary structures structure of the peptides, we utilized both the SOPMA tool (http://npsa-pbil.ibcp.fr/cgibin/npsa_automat.pl?page=/NPSA/npsa_sopma.html) and the Robetta service^81^ (https://robetta.bakerlab.org/) were performed separately. The SOPMA will analyzes and evaluates the target protein structure for alpha helix, beta-turn, random coil, and extended strand formations. The Robetta website is capable of predicting five distinct 3D structures, which are then assessed to optimize the tertiary structure of the protein through the GalaxyRefine server (http://galaxy.seoklab.org/), thus improving the quality of the local structure. The GalaxyRefine modeling method involves ab initio calculation, refining the loop or terminus region in the first-order protein 3D model^82^. However, the rationality of the optimized three-level structure still needs to be verified later.

#### Quality evaluation of tertiary structure model

The SWISS-MODEL online service structural assessment (https://swissmodel.expasy.org/assess), is a homology modeling server that predicts predict the quality of the tertiary structural model. First, the protein that is homologous to the LptD-BTuB mRNA vaccine was located in the ExPDB database to serve as a modeling template^83^. ExPDB is derived from the Protein Data Bank (PDB) (https://www.rcsb.org/) and offers a distinct file for each protein chain^84^. Then, the two sequences were compared and replaced, and the resulting structural model was predicted, evaluated, and refined through repeated steps until achieving a satisfactory model. The final outcomes will be shown in a Ramachandran plot.

In order to enhance the reliability of the results, we utilize verification tools such as Verify 3D, ERRAT to assess the quality of the three-dimensional structure^85^. Meanwhile using ProSA-web server(https://prosa.services.came.sbg.ac.at/prosa.php) to evaluate and explain the potential errors in predicting the tertiary structure of proteins based on the predicted value of Z-score value^86^.

#### Molecular docking

The Toll-like receptor (TLR) family, including Toll-like receptor 4 (TLR-4) (PDB ID: 3FXI), is involved in innate immune response, and can recognize bacterial endotoxin or lipopolysaccharide-induced inflammatory response^87^. To ensure proper binding of the TRL4 immune cell receptor to the LptD-BTuB mRNA vaccine and its subsequent effective immune response, we will simulate the docking of immune molecules^88^. The HDOCK server (http://hdock.phys.hust.edu.cn/) was used to dock the LptD-BTuB mRNA vaccine peptides with TLR-4 (PDB ID: 3FXI). The resulting three-dimensional structure was visualized with PyMOL.

#### Molecular dynamic simulation

The iMOD server was utilized to conduct dynamic simulation analysis of the TLR-4-vacine complexes (http://imods.Chaconlab.org/), which verified the stability and motion of atoms and molecules in the vaccines. These calculations provided us with a better understanding of the atomic-level interactions and conformational changes that take place inside the vaccine when activating the TLR-4 receptor. These insights have set a foundation for the creation of more effective vaccine candidates through rational design. We submitted the PDB file format of LptD-BTuB mRNA, including atomic coordinates. Choose a coarse-grained (CG) model from three options. The Cα atom accounts for the entire residue mass, which can be explained. Then, we selected JAVA in the JSmol plug-in to conserve memory. Finally, the other parameters remain unchanged, and Normal Mode Analysis (NMA) was chosen^89^.

### Supplementary Information

Supplementary Figure 1.

Supplementary Figure 2.

Supplementary Figure 3.

Supplementary Figure 4.

## Data Availability

The data was derived from public domain information: Uniprot database (https://www.uniprot.org/) and PDB library (https://www.rcsb.org/). The data that support the findings of this study are available in the methods and/or supplementary material of this article. The data that support the findings of this study are available from the corresponding author upon request. There are no restrictions on data availability. If you have any questions, please contact me.
