# Supplementary material for: In silico designed novel multi-epitope mRNA vaccines against Brucella by targeting extracellular protein BtuB and LptD
Source: Sci Rep. 2024 Mar 27;14:7278. doi: 10.1038/s41598-024-57793-6 (PMC10973489; doi:10.1038/s41598-024-57793-6)
Supplement: Supplementary file 1 — Supplementary Figure 1. [file 41598_2024_57793_MOESM1_ESM.pdf]

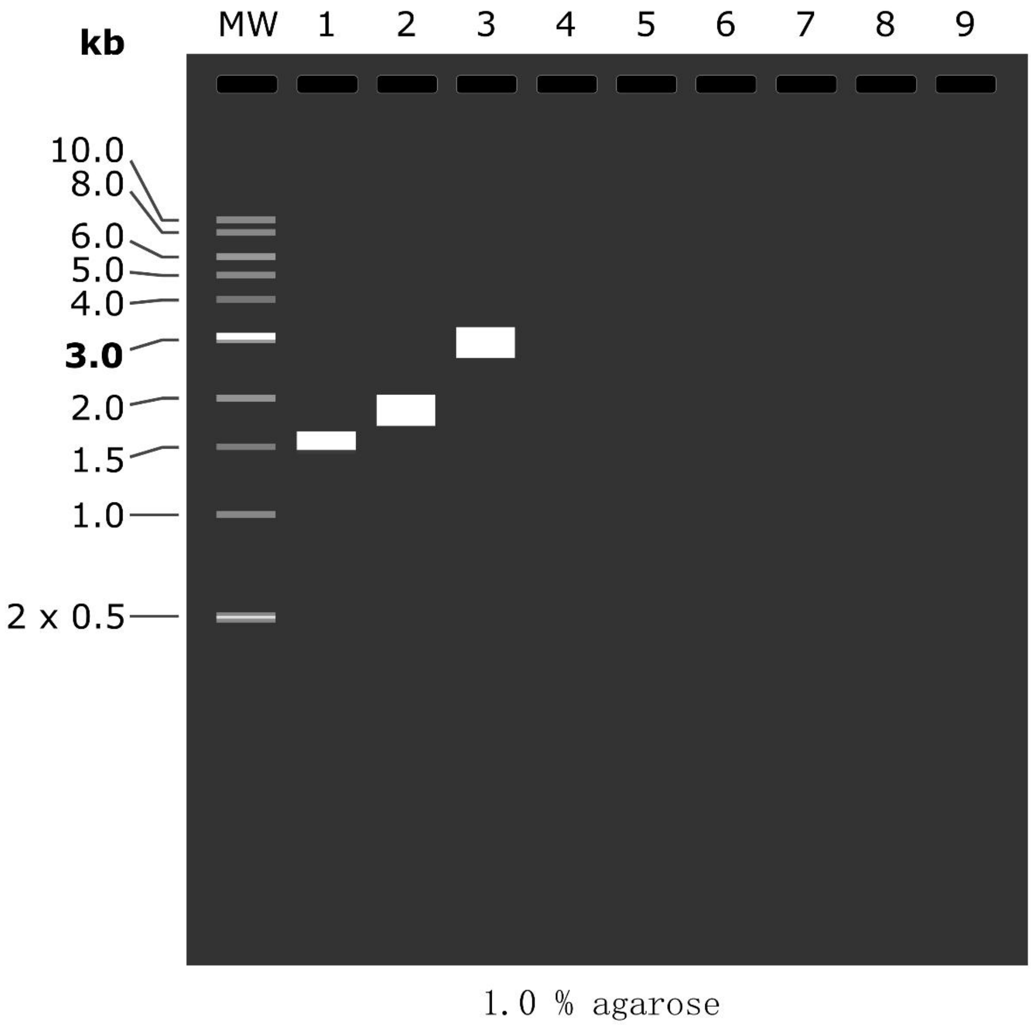

Supplementary Fig. 1 Simulated agarose gel experiment (1.0% agarose). As shown in the figure, “1” stands for LptD-BtuB mRNA vaccine, “2” stands for pVAX1, and “3” stands for recombinant
