# Supplementary material for: In silico designed novel multi-epitope mRNA vaccines against Brucella by targeting extracellular protein BtuB and LptD
Source: Sci Rep. 2024 Mar 27;14:7278. doi: 10.1038/s41598-024-57793-6 (PMC10973489; doi:10.1038/s41598-024-57793-6)
Supplement: Supplementary file 2 — Supplementary Figure 2. [file 41598_2024_57793_MOESM2_ESM.pdf]

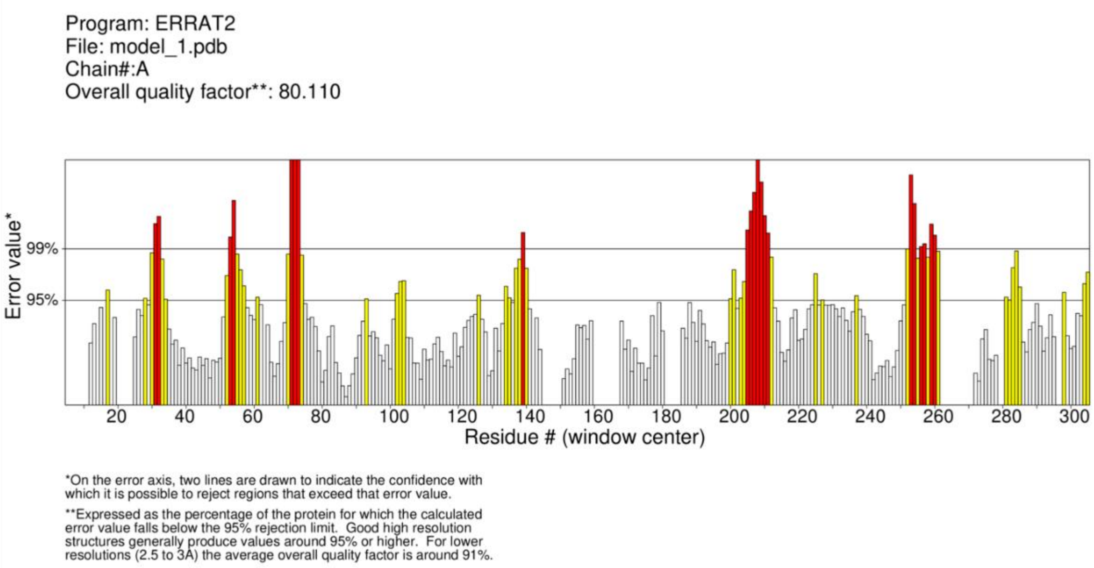

Supplementary Fig.2 ERRAT evaluated the predicted the model of tertiary structure. the ERRAT quality factor of 80.110
