# Supplementary material for: In silico designed novel multi-epitope mRNA vaccines against Brucella by targeting extracellular protein BtuB and LptD
Source: Sci Rep. 2024 Mar 27;14:7278. doi: 10.1038/s41598-024-57793-6 (PMC10973489; doi:10.1038/s41598-024-57793-6)
Supplement: Supplementary file 4 — Supplementary Figure 4. [file 41598_2024_57793_MOESM4_ESM.pdf]

Query Peptides

Peptide ID	Peptide Sequence	SVM Score	Prediction	Hydrophobicity	Hydropathicity	Hydrophilicity	Charge	Mol wt
	MALRGDAIRVDTNFDP	-1.22	Non-Toxin	-0.20	-0.31	0.31	-1.00	1791.23

1/1 50

Query Peptides

Peptide ID	Peptide Sequence	SVM Score	Prediction	Hydrophobicity	Hydropathicity	Hydrophilicity	Charge	Mol wt
	MAYIQTRNPGDEKASH	-0.69	Non-Toxin	-0.30	-1.27	0.34	0.50	1818.22

1/1 50

Query Peptides

Peptide ID	Peptide Sequence	SVM Score	Prediction	Hydrophobicity	Hydropathicity	Hydrophilicity	Charge	Mol wt
	PECIESTNDEPENDEN	-0.26	Non-Toxin	-0.39	-2.04	1.17	-7.00	1835.01

1/1 50

Query Peptides

Peptide ID	Peptide Sequence	SVM Score	Prediction	Hydrophobicity	Hydropathicity	Hydrophilicity	Charge	Mol wt
	PGFSGTNLRFTSEAEW	-1.27	Non-Toxin	-0.12	-0.63	-0.11	-1.00	1799.15

1/1 50

Query Peptides

Peptide ID	Peptide Sequence	SVM Score	Prediction	Hydrophobicity	Hydropathicity	Hydrophilicity	Charge	Mol wt
	QATASTSLDMALYQTR	-0.82	Non-Toxin	-0.18	-0.32	-0.18	0.00	1757.17

1/1 50

Query Peptides

Peptide ID	Peptide Sequence	SVM Score	Prediction	Hydrophobicity	Hydropathicity	Hydrophilicity	Charge	Mol wt
	TYEIQGYNAQTQVSKI	-1.38	Non-Toxin	-0.16	-0.72	-0.24	0.00	1843.27

1/1 50

Query Peptides

Peptide ID	Peptide Sequence	SVM Score	Prediction	Hydrophobicity	Hydropathicity	Hydrophilicity	Charge	Mol wt
	YLPSDKYEDEHPNDDSSR	-0.08	Non-Toxin	-0.46	-2.25	1.01	-3.50	2167.42

1/1 50

Query Peptides

Peptide ID	Peptide Sequence	SVM Score	Prediction	Hydrophobicity	Hydropathicity	Hydrophilicity	Charge	Mol wt
	YSYTMPEPVYGGELNF	-1.65	Non-Toxin	-0.01	-0.45	-0.49	-2.00	1867.29

1/1 50

Supplementary Fig. 4.1 The prediction results of B cell epitope toxicity.

Query Peptides

Peptide ID	Peptide Sequence	SVM Score	Prediction	Hydrophobicity	Hydropathicity	Hydrophilicity	Charge	Mol wt
	ALAPNYDLTL	-1.06	Non-Toxin	0.05	0.44	-0.59	-1.00	1090.38

1/1 50

Query Peptides

Peptide ID	Peptide Sequence	SVM Score	Prediction	Hydrophobicity	Hydropathicity	Hydrophilicity	Charge	Mol wt
	FLQGSFNFAL	-1.21	Non-Toxin	0.17	0.96	-1.09	0.00	1143.45

1/1 50

Query Peptides

Peptide ID	Peptide Sequence	SVM Score	Prediction	Hydrophobicity	Hydropathicity	Hydrophilicity	Charge	Mol wt
	LQTYSGISVK	-0.78	Non-Toxin	-0.05	0.11	-0.40	1.00	1095.41

1/1 50

Query Peptides

Peptide ID	Peptide Sequence	SVM Score	Prediction	Hydrophobicity	Hydropathicity	Hydrophilicity	Charge	Mol wt
	LVYDRDVNTV	-1.14	Non-Toxin	-0.18	-0.06	0.02	-1.00	1193.46

1/1 50

Query Peptides

Peptide ID	Peptide Sequence	SVM Score	Prediction	Hydrophobicity	Hydropathicity	Hydrophilicity	Charge	Mol wt
	NIYMRGMSSK	-1.68	Non-Toxin	-0.26	-0.69	0.01	2.00	1186.55

1/1 50

Supplementary Fig. 4.2 The prediction results of CTL cell epitope toxicity

Query Peptides

Peptide ID	Peptide Sequence	SVM Score	Prediction	Hydrophobicity	Hydropathicity	Hydrophilicity	Charge	Mol wt
	EVGLNWQATASTSLD	-1.51	Non-Toxin	-0.06	-0.19	-0.22	-2.00	1591.91

1/1 50

Query Peptides

Peptide ID	Peptide Sequence	SVM Score	Prediction	Hydrophobicity	Hydropathicity	Hydrophilicity	Charge	Mol wt
	EYDFRIAGIHQLKPE	-1.56	Non-Toxin	-0.20	-0.77	0.27	-0.50	1816.27

1/1 50

Query Peptides

Peptide ID	Peptide Sequence	SVM Score	Prediction	Hydrophobicity	Hydropathicity	Hydrophilicity	Charge	Mol wt
	GDAIRVDTNFDPANA	-0.85	Non-Toxin	-0.18	-0.52	0.31	-2.00	1575.85

1/1 50

Query Peptides

Peptide ID	Peptide Sequence	SVM Score	Prediction	Hydrophobicity	Hydropathicity	Hydrophilicity	Charge	Mol wt
	GLNWQATASTSLDMA	-1.34	Non-Toxin	-0.02	0.01	-0.44	-1.00	1565.93

1/1 50

Query Peptides

Peptide ID	Peptide Sequence	SVM Score	Prediction	Hydrophobicity	Hydropathicity	Hydrophilicity	Charge	Mol wt
	KVTGLEATLSHRFNE	-1.15	Non-Toxin	-0.21	-0.55	0.21	0.50	1702.12

1/1 50

Query Peptides

Peptide ID	Peptide Sequence	SVM Score	Prediction	Hydrophobicity	Hydropathicity	Hydrophilicity	Charge	Mol wt
	NPDLQPEKSRSVEVG	-1.00	Non-Toxin	-0.34	-1.26	0.75	-1.00	1655.01

1/1 50

Query Peptides

Peptide ID	Peptide Sequence	SVM Score	Prediction	Hydrophobicity	Hydropathicity	Hydrophilicity	Charge	Mol wt
	TYEIQGYNAQTQVSK	-1.32	Non-Toxin	-0.22	-1.07	-0.14	0.00	1730.09

1/1 50

Query Peptides

Peptide ID	Peptide Sequence	SVM Score	Prediction	Hydrophobicity	Hydropathicity	Hydrophilicity	Charge	Mol wt
	VGLNWQATASTSLDM	-1.30	Non-Toxin	-0.00	0.17	-0.51	-1.00	1593.99

1/1 50

Query Peptides

Peptide ID	Peptide Sequence	SVM Score	Prediction	Hydrophobicity	Hydropathicity	Hydrophilicity	Charge	Mol wt
	VTGLEATLSHRFNEQ	-1.23	Non-Toxin	-0.18	-0.53	0.02	-0.50	1702.08

1/1 50

Query Peptides

Peptide ID	Peptide Sequence	SVM Score	Prediction	Hydrophobicity	Hydropathicity	Hydrophilicity	Charge	Mol wt
	WPILFSTTSSTHILE	-1.11	Non-Toxin	0.07	0.38	-0.73	-0.50	1732.19

1/1 50

Supplementary Fig. 4.3 The prediction results of HTL cell epitope toxicity
